# A Study on AIN Film-Based SAW Attenuation in Liquids and Their Potential as Liquid Ethanol Sensors

**DOI:** 10.3390/s17081813

**Published:** 2017-08-07

**Authors:** Yong Wang, Zhonggui Xu, Yinshen Wang, Jin Xie

**Affiliations:** The State Key Laboratory of Fluid Power and Mechatronic Systems, Zhejiang University, Hangzhou 310027, China; ywang_mems@zju.edu.cn (Y.W.); xuzhonggui@zju.edu.cn (Z.X.); 21525038@zju.edu.cn (Y.W.)

**Keywords:** AIN, SAW attenuation, liquid ethanol sensors, evaporation rate

## Abstract

In this paper, we report attenuation characteristics of aluminum nitride (AIN) film-based surface acoustic waves (SAWs) in liquids and their potential as liquid ethanol sensors. An AIN film-based SAW resonator was fabricated for liquid sensing application. The fabricated SAW device had a Rayleigh wave mode at a resonant frequency of 147.1 MHz and a low temperature coefficient of frequency (TCF) of −21.7 ppm/K. The signal attenuation in the transmission line of the SAW device was presented when ethanol (ETH) droplets and deionized water (DIW) with different concentrations and volume (0.2–1 µL) were dropped on the sensing area respectively. The attenuation of SAW as a function of time and liquid position was investigated. Residues left on the wave propagation path resulted in a frequency shift of the SAW device after liquid evaporation. For ETH, there was a 49 kHz frequency shift caused by a large amount of residues, while the frequency shift of DIW was not distinct, on account of a clean surface. The linear relationship between evaporation rate and ethanol concentration was demonstrated. The evaporation rate of ethanol droplets showed good consistency, and the evaporation time variation was less than 5% at each concentration level. Therefore, the proposed SAW device had great potentials to determine ethanol concentrations based on evaporation rate.

## 1. Introduction

Over the past few years, sensors based on SAW technology have been extensively explored. In general, the changes in the acoustic wave amplitude and velocity are measured as responses of the SAW sensors. Recently, surface acoustic wave devices have been successfully used in various applications, such as radio frequency (RF) communication [1], chemical and biochemical sensors [2,3], and optical modulators [4]. Nowadays, there has been an increased interest in SAW-based microfluidics applications due to the leakage phenomenon of Rayleigh surface acoustic waves (R-SAWs) in liquids [5,6,7]. The leaky phenomenon can be exploited to speed up biochemical reactions [8], minimize nonspecific bio-bonding [9], and accelerate hybridization rations in protein and DNA analysis [10]. However, R-SAW devices are rarely applied for liquid sensing because of the excessive energy losses experienced at the solid and liquid interface [11]. Moreover, if liquids cover the whole wave propagation path, the power of the SAWs is entirely radiated into the liquids and no signal is detected by receivers.

The Rayleigh wave is composed of longitudinal wave and transversal wave components which induce elliptic displacement of the piezoelectric surface. The longitudinal wave is parallel to the piezoelectric surface and travels along the direction of the wave propagation, whereas the transverse wave component is normal to the wave propagation plane [12]. When the wave propagation path is in contact with liquids, the longitudinal component of R-SAW is coupled into the liquids by the viscosity. Nevertheless, the transversal wave component transmits its energy into the form of a longitudinal compression wave [13], which becomes a leaky surface acoustic wave (LSAW) and dissipates the wave energy into liquids [13,14]. According to wave modes generated on piezoelectric surface, SAW devices can be tailored to different applications. Generally, the shear traversal wave devices are suitable for liquid applications, such as biosensors [15] and liquid concentration sensors [16], whereas R-SAW devices are only for gas-phase operation due to high attenuation [17]. In the past few years, the LSAW devices have been employed for microfluidic pumping [18], mixing [19], microdroplet manipulation [20,21], ejection [22], and particle concentration [23]. In recent studies, SAW devices are used to determine the evaporation rate of different liquids and the amount of residual particles left on the wave path [24], and the phase shift of the SAW device during the evaporation process has also been investigated [25]. However, the detailed attenuation characteristics of R-SAW in liquids have not been frequently reported so far.

There are several methods existing for the detection of liquid ethanol concentration. Srivastava et al. proposed a surface plasmon resonance (SPR) based fiber optic sensor for the detection of water content in ethanol [26]. However, the detection range of ethanol-water mixture was only 0–10% (*v*/*v*). Lee et al. fabricated a chemical sensor based on conductive ZnO nanorod arrays for the detection of ethanol solution at room temperature [27]. The ethanol concentrations were measured by using electrical impedance spectroscopy (EIS), whereas the fabricated processes of ZnO nanorod arrays were complicated. Lindner et al. proposed a versatile acoustic waveguide ethanol sensor with a sensing scheme to measure the variation of the wave velocity in different concentrations of solutions [28]. By measuring the time shift of the receiver signal, they determined ethanol concentrations. Nevertheless, the amount of liquids used for sensing was too large.

AIN films are stable in liquid solutions and have low insertion loss, so they are good candidates for liquid sensing. In this paper, we fabricated an AIN film-based SAW resonator to produce Rayleigh waves, and investigated the attenuation of R-SAW when it encountered a droplet applied at the center of the wave propagation path. The response of the Rayleigh wave in the presence of liquids covering on the wave path was detected by a two-port SAW resonator due to its low insertion loss and high signal stability. Both DIW and liquid ethanol were used as target liquids. The detailed attenuation characteristics of R-SAW as a function of liquid type, volume, evaporation time, concentration and the position of the liquid dropped were investigated theoretically and experimentally. The experimental results showed that the energy of R-SAW was dissipated into liquids. The bigger the sensing area covered by the liquids, the more energy leaked into the liquids. Good linearity was found between evaporation time and ethanol concentration. Based on this finding, we proposed an AIN film-based SAW device to determine ethanol concentrations by measuring evaporation rate. The proposed method has merits of small scale, simple implementation and large detecting ranges (10–90%Vol). Meanwhile, the amount of the liquids used for sensing is quite small, so the SAW device is useful for quick detection of ethanol concentrations.

## 2. Experimental

### 2.1. Theoretical Analysis

When an incident SAW is propagating to a droplet, part of the incident SAW is scattered by air-liquid-solid contact line, while the rest are penetrated into the area covered by the droplet. The penetrated SAW attenuates and changes its mode to a leaky SAW upon its arrival at the boundary between solid and liquid, due to the mismatch in acoustic velocities between solid and liquid [13]. The attenuation is induced to both longitudinal and transverse wave components. Generally, the attenuation coefficients for the longitudinal wave $\alpha_{L}$ and transverse wave $\alpha_{T}$ can be estimated as [29]:

(1) $$\alpha_{L}=\frac{\sqrt{\rho_{f}\eta_{f}\omega^{3}}}{4\sqrt{2}\pi^{2}\rho_{s}v_{s}^{2}}$$

(2) $$\alpha_{T}=\frac{\rho_{f}v_{f}}{\rho_{s}v_{s}}\frac{1}{\lambda }$$

where $\rho_{f}$, $v_{f}$, $\rho_{s}$, $v_{s}$, λ, ω=2πf and $\eta_{f}$ are the density of the liquid, the sound velocity in liquids, the density of the substrate, the SAW velocity in the substrate, the wavelength of the acoustic wave, the angular frequency, and the dynamic viscosity of the liquid, respectively.

**Figure 1:**
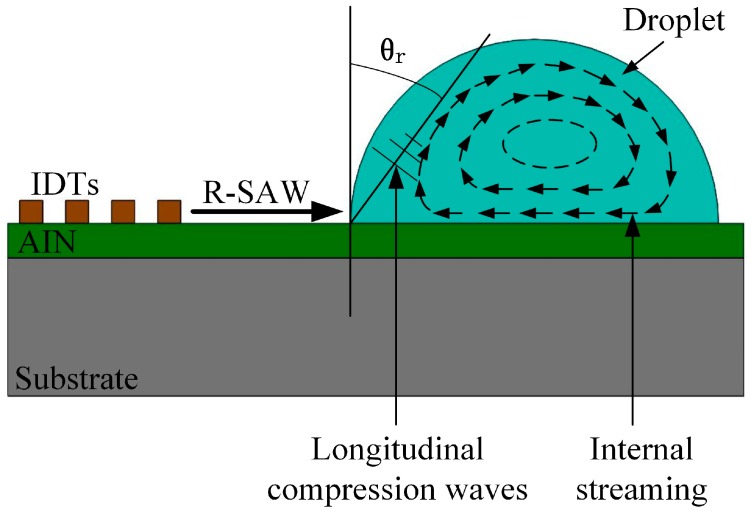
Interaction between Rayleigh wave and a droplet dropped on the wave propagation path.

Since the SAW velocity in the substrate (4708 m/s) is much larger than the value in water (1498 m/s), the energy of longitudinal wave component penetrated into the liquid is much smaller than the transverse wave component [30]. Therefore, the attenuation is mainly contributed by a transverse wave. As shown in Figure 1, the transverse component of R-SAW transmits its energy into liquids in the form of a longitudinal compression wave at a specific angle, known as the Rayleigh angle $\theta_{r}$ [13]:

(3) $$\theta_{r}=\sin^{-1}\left(\frac{v_{f}}{v_{s}}\right)$$

where $v_{f}$ and $v_{s}$ are the velocities of the acoustic wave in the liquid and substrate.

When a droplet is dropped on the wave propagation path, the energy of SAWs is radiated into liquids. The SAW propagation improves continuously due to the decrease of the area covered by the liquids. After liquid evaporation, there is some residue left on the wave path because of the incomplete volatilization of impurities existing in the liquids, like obstacles, which result in the frequency shift and signal attenuation of the SAW device. The change in surface mass density of the SAW device after the liquid evaporation can be defined as:

(4) $$\Delta \rho_{s}=\frac{\Delta m}{A}$$

where Δm is the change in surface mass, A is the area covered by residues, and $\Delta \rho_{s}$ is the change of surface mass density of the piezoelectric substrate.

The change in resonant frequency of the SAW device due to mass loading (neglecting the viscoelastic and electric effects) had been characterized by the classic Sauerbrey equation [31]:

(5) $$\Delta f_{0}=-\frac{2f_{0}^{2}}{\sqrt{\rho_{s}\mu_{s}}}*\Delta \rho_{s}$$

where $f_{0}$ is the resonant frequency of the SAW device, $\Delta \rho_{s}$ is the change in surface mass density of the piezoelectric substrate, $\rho_{s}$ is the density of the piezoelectric substrate, and $\mu_{s}$ is the substrate shear modulus.

### 2.2. Device Design and Fabrication

The proposed SAW device has a large area of 9100 × 5200 µm^2^, with 50 pairs of interdigital transducer (IDT) fingers. The width of the fingers is 8 µm, and this results in a resonant frequency of 147.1 MHz. The acoustic aperture is 2400 µm in width to avoid diffraction. The IDTs are surrounded by a total of 200 reflectors gratings (100 on each side) with a 16 µm pitch to create a standing wave pattern between IDTs. Other parameters of the SAW device are listed in Table 1.

**Figure 2:**
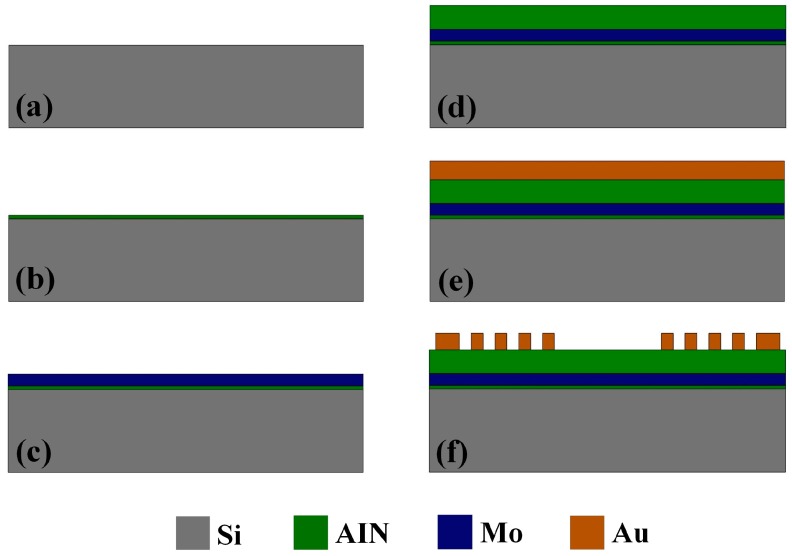
Fabrication process flow of the SAW device.

**Figure 3:**
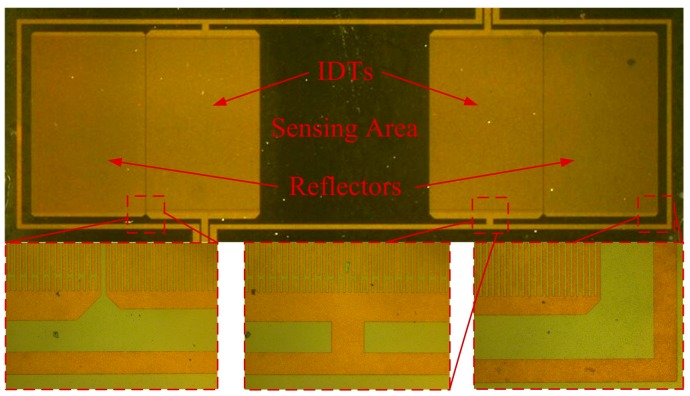
Optical micrograph of the fabricated SAW device.

The fabrication process flow started with a 450 µm Si substrate, as shown in Figure 2. At first, a 20 nm AIN seed layer was deposited on the Si substrate in order to obtain good texture in the piezoelectric AIN film. The AIN seed layer also acted as an insulating layer. Then, a 0.2 µm Mo layer was deposited as the bottom electrode of the resonator. After that, a 1.3 µm AIN piezoelectric layer was deposited on the Mo layer by sputtering. Finally, a 300 nm Au film was deposited on the AIN film and patterned by ion etching to form the IDTs. The fabricated SAW device is shown in Figure 3.

### 2.3. Characterization of AIN Film

**Figure 4:**
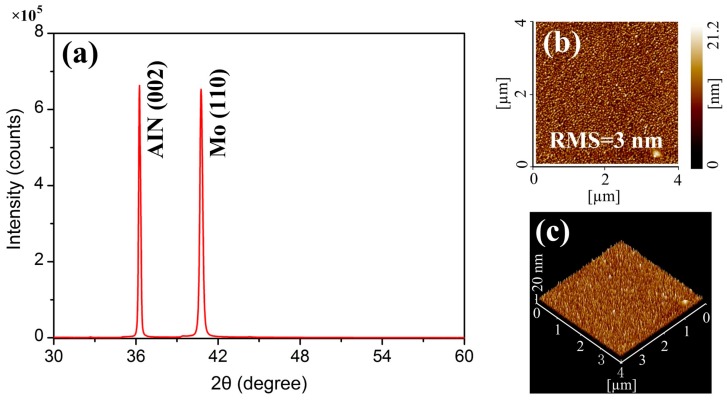
(**a**) X-ray diffraction (XRD) spectrum of the aluminum nitride (AIN) film on the Mo bottom electrode; (**b**) and (**c**) Atomic force microscopy (AFM) images of the deposited AIN film.

Figure 4a shows the X-ray diffraction (XRD) pattern of an AIN film on the Mo bottom electrode with a large peak at the angle of 36.26° corresponding to a good (002) crystal orientation. A higher XRD intensity indicates better crystallite quality and fewer defects. The full width at half maximum (FWHM) of XRD curve of the deposited AIN film was 0.172°, indicating a small angular dispersion of the crystallites around the *c*-axis. The mean grain size was calculated by the Debye-Scherrer formula [32]:

(6) $$D=\frac{C\lambda_{x}}{\beta \cos\theta }$$

where C is the shape factor of average crystallite with a value of 0.94, $\lambda_{x}$ is the X-ray wavelength (1.5405 Å for the Cu target), β is the FWHM in radians, and θ is the Bragg angle. The calculated mean grain size was about 50.8 nm. Figure 4b,c illustrate the morphology and surface roughness of the film measured by atomic force microscopy (AFM). The root mean square (RMS) roughness of the film was about 3 nm over an area of 4 × 4 µm^2^.

The quality factor (*Q*) is a key parameter to evaluate the performance of both the resonators and the resonator-based applications. Here, we used a called the “S_21_–S_11_ magnitude method” to extract the value of *Q* [33,34]. Because the wave propagation path between input IDTs and output IDTs was too long (2400 µm) for a resonator, the Q value of the fabricated SAW device was only approximately 190.

### 2.4. Experimental Set-Up

**Figure 5:**
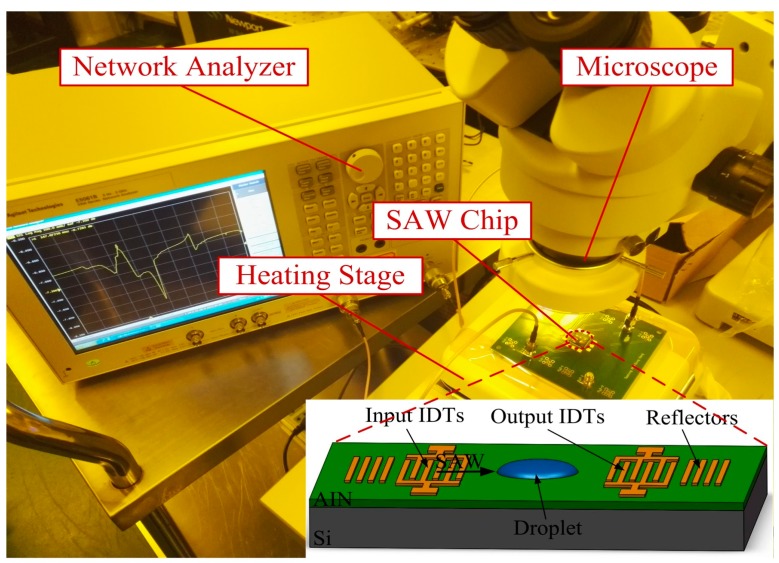
Measurement set-up of the SAW device for evaluating the attenuation of Rayleigh surface acoustic waves (R-SAWs) in liquids.

The experimental set-up is illustrated in Figure 5. A two-port AIN film based SAW resonator was employed for liquid sensing tests with a droplet dropped on the wave path. A network analyzer (Agilent E5061B) with the maximum power of 5 dBm was connected to the SAW resonator to produce Rayleigh waves. Since the variation of temperatures makes a great difference to liquid evaporation, the sensor was heated by a heating stage which could control the temperature invariably at 25 °C. A small droplet applied for test was generated by a 1 µL microsyringe. In order to precisely control the liquid position on the wave propagation path, the operation was conducted under a microscope. The evaporation process mainly depends on physical properties, such as evaporation area, vapor pressure, surface tension, temperature and so on. Therefore, all experiments were operated at the same room conditions of atmospheric pressure, 45% RH, and 25 °C. Before each experiment, the surface of the SAW device was cleaned by DIW in order to have the same initial signal.

## 3. Results and Discussion

When a droplet is applied on the wave propagation path, the energy of R-SAW is radiated into liquids, which results in the attenuation of the SAW device. The energy of the longitudinal wave radiated into the liquids can produce an acoustic radiation force in the direction of the sound propagation [35], which leads to an internal acoustic streaming inside a droplet. Acoustic streaming may cause the shape deformation of the droplet and has an impact on wave attenuation and liquid evaporation. However, since the wavelength of the fabricated SAW device is much smaller than the conventional microfluidic devices and the power applied to the input IDTs is only 5 dBm, there is only minor internal acoustic streaming inside a droplet. In other words, the effect of the acoustic streaming on droplet shape and contact angle can be neglected. In addition, we also considered the influence of back reflection of the acoustic wave on wave attenuation. When an incident SAW propagated into a droplet, a part of the incident SAW was scattered by the air-liquid-solid contact line. There is some research on the back reflection of the sound waves radiated into the droplet [29,35]. The experimental results indicated that about 5–10% of the incident energy hitting on the droplet was scattered away by a water droplet. This was quite small in comparison with the energy radiated into liquids, so its influence on wave attenuation was also neglected in this study.

**Figure 6:**
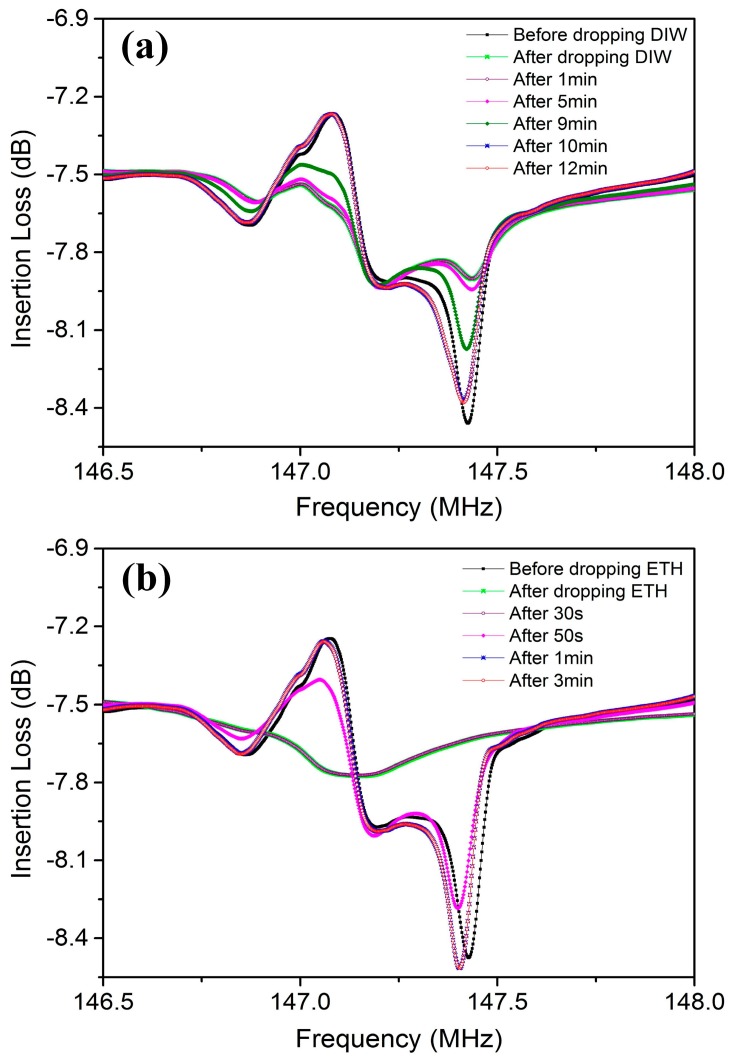
The changes in attenuation of the SAW device when (**a**) deionized water (DIW) and (**b**) ethanol (ETH) are dropped on the center of the wave path respectively.

**Figure 7:**
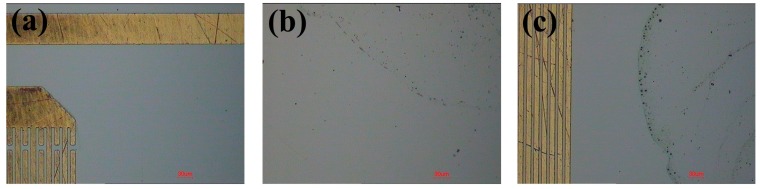
Micrograph of the residues left on the wave propagation path (**a**) before, and after (**b**) DIW and (**c**) ETH are evaporated.

Figure 6 reports the changes in attenuation of R-SAW when 1 µL DIW and liquid ethanol were dropped on the center area of the wave propagation path. When a droplet was applied on the surface of the SAW device, the R-SAW attenuated rapidly, and the signal returned to its initial level only when the liquids evaporated completely. The deionized water dropped on the solid substrate had an initially spherical shape, and the contact angle was approximately 90°, while the shape of liquid ethanol was not spherical due to a weak surface tension, and the contact angle was only 30°. For 1 µL DIW and liquid ethanol, the diameter of contact area between liquids and solid surface was approximately 1620 µm and 3200 µm respectively. Since the wave propagation length between input IDTs and output IDTs was 2400 µm, there were some ethanol droplets flowing onto the surface of the IDTs and outside of the acoustic aperture, which resulted in a higher attenuation of the SAW device, as shown in Figure 6b. As illustrated in Figure 6a, the evaporation rate of DIW was slower than ethanol, due to a high surface tension between water molecules. After liquid evaporation, there was some residue left on the wave path, due to incomplete volatilization of impurities in liquids, which resulted in a frequency shift and small signal noise in the SAW device. For liquid ethanol, the frequency shift of the SAW device was 49 kHz, as a result of a large amount of residual particles left on the wave path, as shown in Figure 7c. However, the frequency shift of DIW was not distinct because of a clean surface, as shown in Figure 7b.

**Figure 8:**
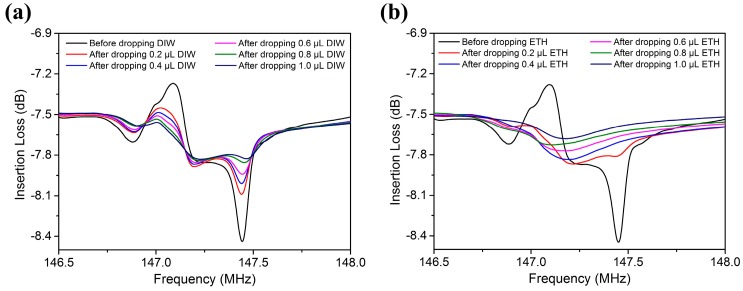
The attenuation of R-SAW in different volumes of (**a**) DIW and (**b**) ETH after dropping them on the wave propagation path.

We also investigated the volume effects of DIW and liquid ethanol on wave attenuation, as shown in Figure 8. The attenuation length of the Rayleigh wave propagating into liquids can be calculated by:

(7) $$L_{SAW}=\frac{1}{\alpha_{L}}=\frac{\rho_{s}v_{s}\lambda }{\rho_{f}v_{f}}=\frac{\rho_{s}v_{s}^{2}}{\rho_{f}v_{f}f_{0}}$$

where $\rho_{f}$ and $\rho_{s}$ are the density of the liquid and substrate respectively, $v_{f}$ is the velocity of SAW in liquids, $v_{s}$ is the SAW velocity in the substrate, λ is the wavelength, $f_{0}$ is the resonant frequency of the SAW device, and $\alpha_{L}$ is the attenuation coefficient of the Rayleigh wave.

The penetration depth of the longitudinal component of the Rayleigh wave into liquids can be determined by [36]:

(8) $$\delta =\sqrt{\frac{2\eta }{\rho_{f}w}}$$

where w is the angular frequency, and η and $\rho_{f}$ are the dynamic viscosity and density of the liquid.

The Rayleigh angle $\theta_{r}$ = 18.6° was calculated by Equation (3). The SAW velocity in the AIN/Si substrate is approximately 4708 m/s, and the sound velocity in water is 1498 m/s. Therefore, the attenuation length of R-SAW in water was approximately *L_SAW_* = 357 µm, which can be calculated by Equation (7). From Equation (8), we determined that the penetration depth of R-SAW in water was about 50 nm.

When a droplet is dropped on the solid substrate, the contact area has a circular shape. The diameter of the contact area was measured by a microscope. The measurements are listed in Table 2. As the attenuation length of R-SAW in liquids was smaller than the contact diameter of DIW or liquid ethanol (0.2–1 µL), it could not pass through the area covered by these liquids. However, R-SAW could propagate along the external boundary of DIW covered on the wave path, because the contact diameter between water and the solid surface was smaller than the acoustic aperture (2400 µm). As a result, the larger the area covered by liquids, the more the energy of R-SAW radiating into them, as shown in Figure 8a. If liquids covered the entire wave propagation path, the energy of R-SAW would leak entirely into the liquids, and the signal would not be detected by receivers, as shown in Figure 8b. In tests, the contact diameter between 1 µL DIW and solid surface was subequal to 0.2 µL liquid ethanol, and the attenuation of R-SAW in two kinds of liquids is also nearly the same. Meanwhile, the penetration depth of R-SAW in water was approximately only 50 nm, which was far less than the droplet height. Therefore, we could conclude that the attenuation of R-SAW in liquids may depend critically on the diameter of the contact area, but would be less correlated with the droplet height. If the surface of the SAW device were treated by hydrophobic materials, the contact diameter would decrease and then result in less energy radiating into the liquids.

**Figure 9:**
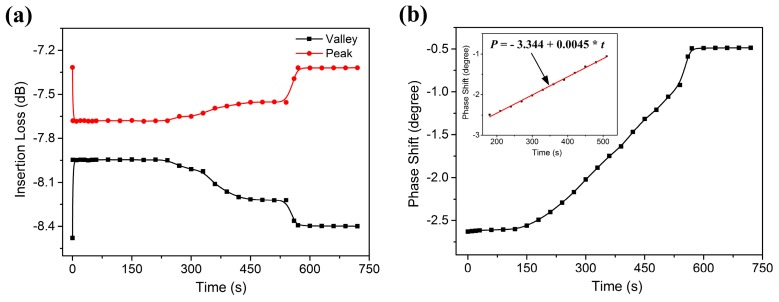
The changes in (**a**) attenuation amplitude and (**b**) phase shift versus time of the SAW device during the entire DIW evaporation process.

The changes in attenuation amplitude of the SAW device versus time during the DIW evaporation process are illustrated in Figure 9a. There are two basic evaporation modes existing in the liquid evaporation process [37]. One is the constant contact radius (CCR) mode, where the contact area remains constant while the contact angle decreases with time [38]; the other is the constant contact angle (CCA) mode, where the contact area shrinks with time while the contact angle remains constant [39]. Liquid evaporation mostly occurs through either one of these two modes, or by a combination of the two.

Three distinct stages were observed during the DIW evaporation process. In the first stage, when 1 µL DIW was applied on the wave path, the amplitude of R-SAW attenuated rapidly as a result of the energy of the acoustic wave radiating into liquids. As the droplet evaporated, the contact angle and height of the droplet decreased with time, while the contact area between the droplet and solid substrate remained constant. As mentioned above, the attenuation of R-SAW depended mostly on the diameter of the contact area, but was less correlated to liquid height. Consequently, the energy of R-SAW radiated into liquids was nearly constant in this stage, and then resulted in a relative small change in attenuation amplitude. In the second stage, since the surface tension of the water molecules was larger than interfacial tension between the solid substrate and the liquid, the contact area shrank with time. Therefore, the attenuation of the R-SAW decreased slowly with evaporation time. Finally, when the droplet was nearly evaporated, there was only a thin layer of water film left on the wave propagation path. The sudden break of the film caused a transient recovery of the sensor and the signal returned to its original level only when small droplets were evaporated completely. The phase variation of the SAW device during the entire evaporation process of DIW is shown in Figure 9b. The phase variation could also be divided into three stages, but the phase shift had an approximate linear relation with evaporation time at some specific time periods.

**Figure 10:**
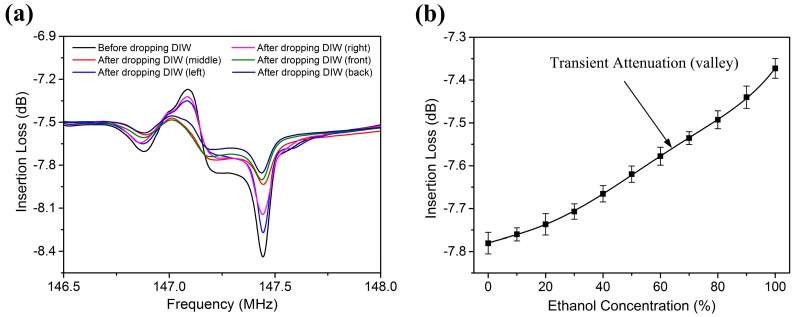
(**a**) The attenuation of the SAW device before and after 1 µL DIW is dropped in different positions of the wave path; (**b**) The transient attenuation of the SAW device after dropping different concentrations of liquid ethanol.

The propagation of R-SAW was strongly affected by positions of the liquid dropped. In order to study the effects of liquid position on wave attenuation, the droplets were dropped in the middle, left, right, front and back of the center sensing area, respectively. As shown in Figure 10a, the signal of the SAW device had a higher attenuation when a droplet was dropped on the center area of the wave propagation path. The energy of SAW was focused mostly in the middle of the wave path, and a small amount of energy was diffracted to the outside of the acoustic aperture. Since the attenuation of the SAW device with liquids dropped on the sides of the center sensing area was nearly the same, we could deduce that the wave propagation was symmetric. When the droplets were dropped in the middle of sensing area near input IDTs or output IDTs, the attenuation of R-SAW was almost the same. A relatively small difference in attenuation amplitude between the positions near input IDTs and output IDTs may be caused by energy transmission loss during the wave propagation process. Therefore, it could be concluded that the Rayleigh angles radiated into liquids were approximately equal along the middle of the wave propagation path.

As mentioned above, the R-SAW attenuated rapidly when a droplet was introduced to the wave path. Figure 10b shows the transient attenuation of the Rayleigh wave to different concentrations of liquid ethanol. For ethanol, the covered area by liquids increased with ethanol concentration, due to the decrease of surface tension among ethanol molecules. Therefore, the transient attenuation of the SAW device had an increasing tendency with the increment of ethanol concentration.

**Figure 11:**
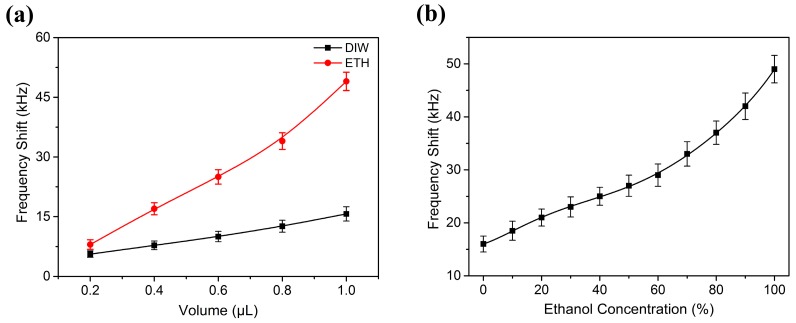
Resonant frequency shift of the SAW device in response to (**a**) liquid volume and (**b**) liquid ethanol concentration after their evaporation.

After the liquid evaporation, there was some residue left on the wave path, which resulted in a frequency shift of the SAW device. The residues still existed on the surface of the SAW device even after the device had been placed in a clean room for a long time, so the constitution of the residues might be incomplete volatilization of impurities in liquids. The frequency shift of the SAW device to a different volume of DIW and liquid ethanol after their evaporation is illustrated in Figure 11a. The experimental results showed that the frequency shift had an approximate linear relationship with liquid volume, because of more residual particles left on the wave propagation surface with the increment of liquid volume. Compared to the same volume DIW, more ethanol residues were left on the wave path, which resulted in a higher frequency shift. When the ethanol droplet was nearly evaporated, a thin liquid ethanol film formed. The sudden break of the liquid film generated many small ethanol droplets. The distribution of small ethanol droplets was chaotic, which resulted in the perturbation of the frequency shift. However, the increasing trend between frequency shift and ethanol concentration was apparent, due to more residues left on the solid surface with a high concentration after the ethanol evaporation, as shown in Figure 11b.

**Figure 12:**
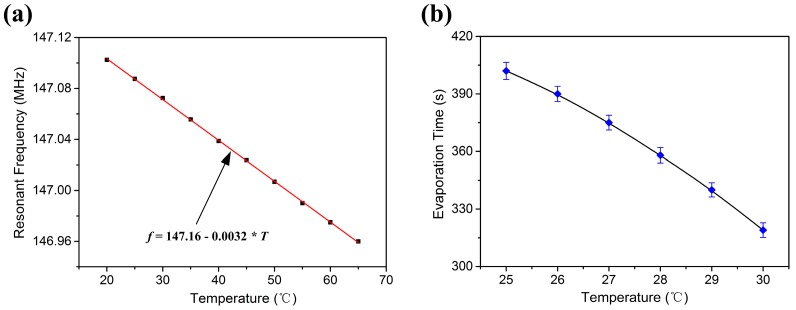
(**a**) Resonant frequency of the SAW device as a function of temperature; (**b**) Evaporation time variation of 0.6 µL DIW relative to temperature.

It is well known that the propagation surface of the SAW device is heated when a droplet is applied on the wave propagation path [40]. Some studies show that acoustic heating and dielectric heating exist simultaneously in heating process of the wave propagation surface [41]. Nevertheless, acoustic heating plays a more important role in heating of the substrate. In addition, we observed the acoustic streaming phenomenon during the liquid evaporation process. Therefore, the temperature rise of the substrate was mainly caused by an acoustic heating effect. The temperature rise of the piezoelectric surface may influence the performance of the sensor and liquid evaporation. However, the sensor employed for liquid sensing in this study had a very small TCF of −21.7 ppm/K, as shown in Figure 12a. Furthermore, the maximum power applied for the input IDTs was only 5 dBm, so there was only a minor temperature rise for the piezoelectric surface. Therefore, the resonant frequency shift caused by the temperature rise was very small. In other words, the frequency shift of the SAW device was contributed mainly by the residual particles left on the wave path after liquid evaporation. Figure 12b shows the evaporation time variation as a function of temperature during the evaporation process of 0.6 µL DIW. As the temperatures rise, the evaporation rate speeds up and the evaporation time decreases.

**Figure 13:**
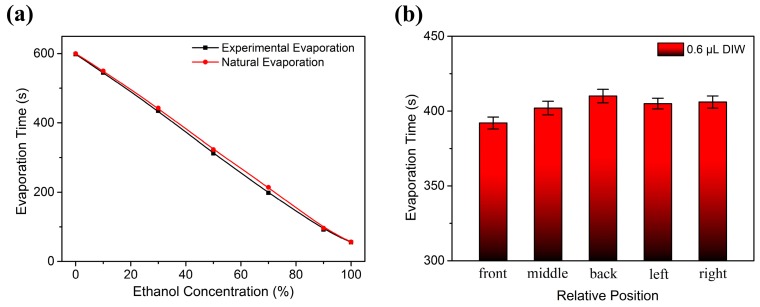
(**a**) Evaporation time as a function of liquid ethanol concentration whether the power is applied at the input IDTs; (**b**) Evaporation rate in response to relative position of the liquids.

Figure 13a reports the evaporation time variation of different concentrations of liquid ethanol (0.6 µL) whether the power is applied at the input IDTs. The evaporation rate sped up when the power was connected to the input IDTs, which indicates that there was a temperature rise (0.5–0.6 K) on the wave propagation surface. In order to identify surface temperature distribution of the SAW device, the same volume droplets were dropped in different positions of the center sensing area respectively. The evaporation tests were repeated many times. The experimental results indicated that the temperature near the input IDTs was a little higher than the position next to the output IDTs, due to more energy from R-SAWs being radiated in the liquids, as shown in Figure 13b. Besides, the center sensing surface had slightly higher temperature than the positions on the sides of the center sensing area, which indicated that the acoustic wave propagated with the highest intensity in the middle of the wave propagation path.

**Figure 14:**
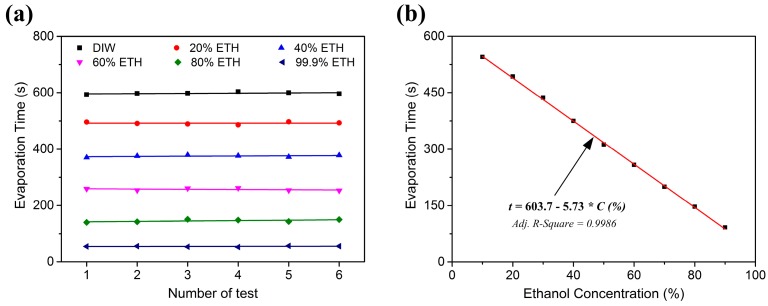
(**a**) Stability responses of the evaporation rate to different concentrations of liquid ethanol; (**b**) Evaporation time as a function of ethanol concentration for a large detection range.

Figure 14a shows the stability of the evaporation rate to different concentrations of liquid ethanol. The sensor was repeatedly tested at fixed ethanol concentrations from 0% to 99.9% (*v*/*v*), at intervals of 20%Vol. The experimental results show that the evaporation rate shows a good consistency and evaporation time variation was less than 5% at each concentration level. The linear relationship between evaporation rate and ethanol concentration was observed, as shown in Figure 14b. The evaporation time showed good linearity for a large concentration range from 10% to 90% (*v*/*v*). Therefore, we firstly propose an AIN film based SAW ethanol sensor with a sensing scheme to measure evaporation rate. The sensitivity of the ethanol sensor is defined as:

(9) $$S=\frac{\Delta t}{\Delta C}$$

where Δt and ΔC are the changes in evaporation time and ethanol concentration.

The sensitivity of the sensor was up to 57.3 s/10% (*v*/*v*), if the SAW device was used to measure evaporation rate. Meanwhile, the evaporation tests had merits of simple implementation and good repeatability. Therefore, the SAW device could be used as a liquid ethanol sensor by measuring evaporation rates.

## 4. Conclusions

An AIN film-based SAW resonator was fabricated and characterized for liquid sensing applications. The attenuation characteristics of R-SAW in different volumes and concentrations of DIW and liquid ethanol were studied. The experimental results showed that R-SAW attenuated rapidly when it encountered liquids. If the whole wave propagation path was covered by liquids, no signal would be detected at the output IDTs. When the liquids were nearly evaporated, there was a signal jump, due to the sudden break of the thin liquid film. After liquid evaporation, there was some residue left on the wave path, which resulted in a frequency shift by the SAW device. The attenuation of R-SAW in liquids depended mostly on the contact area covered by liquids, but was less correlated with liquid height. The acoustic heating phenomenon and temperature distribution of the surface of the SAW device were investigated. The farther the distance away from input IDTs, the lower the temperature. A good linear relationship was found between ethanol concentration and evaporation rate. An AIN film-based SAW ethanol sensor was proposed by measuring evaporation rate. The proposed method has merits of small scale, simple implementation, and large detecting ranges [10–90% (*v*/*v*)].

## Figures and Tables

**Table 1:** The design parameters of the surface acoustic wave (SAW) device.

Parameter	Value
Wavelength λ (µm)	32
interdigital transducer (IDT) thickness *h* (nm)	300
Acoustic aperture *W* (µm)	2400
Delay line length *L* (µm)	2400
Finger pairs *N_IDT_*	50 × 50
Grating number *N_r_*	200

**Table 2:** The contact diameter between different volume liquids and piezoelectric surface.

Volume (µL)	Contact Diameter (µm)	
	DIW	Ethanol
0.2	880	1630
0.4	1080	1980
0.6	1320	2360
0.8	1480	2850
1	1620	3200
